# Factors associated with smoking cessation in Chinese psychiatric professionals: A cross-sectional survey

**DOI:** 10.18332/tid/189299

**Published:** 2024-06-13

**Authors:** Long Chen, Wenzheng Li, Song Wang, Mengyue Gu, Feng Jiang, Huanzhong Liu, Yi-lang Tang

**Affiliations:** 1 Department of Psychiatry, Chaohu Hospital of Anhui Medical University, Hefei, China; 2 Anhui Clinical Center for Mental and Psychological Diseases, Hefei Fourth People’s Hospital, Hefei, China; 3 Affiliated Psychological Hospital of Anhui Medical University, Hefei, China; 4 School of International and Public Affairs, Shanghai Jiao Tong University, Shanghai, China; 5 Institute of Healthy Yangtze River Delta, Shanghai Jiao Tong University, Shanghai, China; 6 Anhui Provincial Key Laboratory for Brain Bank Construction and Resource Utilization, Anhui Medical University, Hefei, China; 7 Anhui Psychiatric Center, Anhui Medical University, Hefei, China; 8 Department of Psychiatry and Behavioral Sciences, Emory University, Atlanta, United States; 9 Joseph Maxwell Cleland Atlanta Veterans Affairs Medical Center, Decatur, United States

**Keywords:** smoking cessation, Chinese psychiatric professionals, associated factors, burnout, gender and smoking

## Abstract

**INTRODUCTION:**

Healthcare workers are integral to public smoking cessation; however, their own smoking behavior can create a significant obstacle to intervening in patients' cessation efforts. Conversely, their success in quitting can enhance their ability to support patients. Research on smoking behavior, particularly smoking cessation among Chinese psychiatric professionals is limited. This study addresses this gap by examining the factors associated with smoking cessation in this population, providing insights for targeted tobacco control policies.

**METHODS:**

A cross-sectional survey was conducted, targeting psychiatric professionals including psychiatrists and psychiatric nurses, in 41 tertiary psychiatric hospitals in China. From January to March 2021, a WeChat-based questionnaire was distributed to collect demographic, occupational, and health-behaviors (including smoking) data. Statistical analyses, including the chi-squared test and adjusted binary logistic regression analysis, were conducted to identify the factors associated with smoking cessation.

**RESULTS:**

Among the 12762 psychiatric professionals who participated in the survey, 11104 (87.0%) were non-smokers, 1196 (9.4%) were current smokers, and 462 (3.6%) were ex-smokers. Several factors were found to be associated with smoking cessation. Women had a higher prevalence of ex-smokers than men (AOR=1.88; 95% CI: 1.332–2.666, p<0.001). Compared to East China, the prevalence of ex-smokers among participants in Central and Northeast China was lower. Older age (≥50 years), higher level of education (Master’s degree or higher), and non-drinkers, showed a higher likelihood of being ex-smokers. Notably, compared to current smokers, ex-smokers reported a lower prevalence of burnout (AOR=0.70; 95% CI: 0.552–0.892, p=0.004).

**CONCLUSIONS:**

Smoking cessation interventions or health promotion programs should also focus on gender, age, education level, region, alcohol use, and burnout to effectively address smoking cessation within this specific professional group.

## INTRODUCTION

Smoking is a major public health problem, with an estimated 26.6% of adults aged ≥15 years in China being current smokers according to the World Health Organization (WHO)^1^. Smoking can directly or indirectly cause numerous diseases and increase mortality risk, emerging as the leading risk factor for global cancer deaths and disability-adjusted life-years (DALYs)^2,3^. In the 2010s, about 20% of Chinese adult male deaths were attributed to smoking^4^. Without effective smoking cessation, the annual number of deaths caused by tobacco in China will rise from about 1 million in 2010 to 2 million in 2030, and 3 million in 2050^4^.

Despite the well-documented health hazards associated with smoking, many medical professionals, including psychiatrists and nurses, start or persist in smoking. A recent review covering 246 studies and 497081 doctors showed that the smoking rate was 17% among psychiatrists^5^. Another study on cigarette smoking among mental health professionals found that 16.1% of psychiatrists and 10% of psychiatric nurses were smokers, respectively^6^. A survey on the smoking status of nurses in psychiatric and general hospitals in China found that the lifetime prevalence of smoking among psychiatric nurses in China was 14.5% and the current prevalence of smoking was 13.4%, which was significantly higher than that of non-psychiatric nurses (1.2% and 1.2%)^7^. Studies suggested smoking physicians were less likely to offer patients smoking cessation interventions^8^. Similarly, nurses are recognized as role models for non-smoking. Nurses who do not smoke are likely to be more effective in supporting patients to quit than nurses who smoke^9^.

Tobacco control, adopted globally, holds the potential to significantly reduce the risk of smoking-related diseases. Smoking cessation, especially at a young age, is significantly associated with the reduction of relative excess mortality^10,11^ and improved mental health including reduced depression, anxiety, stress, and quality of life compared with continuing to smoke^12^, and smoking cessation should be a priority of mental health services. Medical professionals, positioned as guardians and promoters of health, play a crucial role in asking about smoking status and providing interventions. Highlighting this, research shows that the smoking rate of psychiatric patients is higher than the general population, possibly due to low education, substance abuse, and inattention by healthcare providers^13^. In this context, the role of psychiatrists and psychiatric nurses in identifying smoking in patients and providing effective interventions becomes even more crucial than their non-psychiatric counterparts. Given this high prevalence and the influence psychiatrists and psychiatric nurses have on their patients’ smoking cessation efforts, it is important to characterize the smoking cessation behavior of psychiatrists and psychiatric nurses.

Various factors have been reported as contributors to smoking behavior or the success or failure of smoking cessation. Common factors associated with smoking behavior include age (older age group), sex (male), professional title, unhealthy behavior (such as alcohol use or poor sleep), as well as poor job satisfaction and burnout^6,14-17^. On the other hand, several factors have been reported to be associated with smoking cessation success, including receiving and accepting a doctor’s recommendation to stop smoking, possessing a higher level of education, engaging in regular exercising, and benefiting from smoking prohibitions in public places^18,19^. Understanding this intricate interplay of factors is essential for developing effective strategies to address smoking behavior and enhance smoking cessation outcomes.

To date, no national study in China has investigated factors associated with smoking cessation among psychiatric professionals. This cross-sectional study among Chinese psychiatric professionals seeks to investigate sociodemographic and mental health differences between active and former smokers that may have impacted smoking cessation efforts.

## METHODS

### Study design and participants

**Figure 1:**
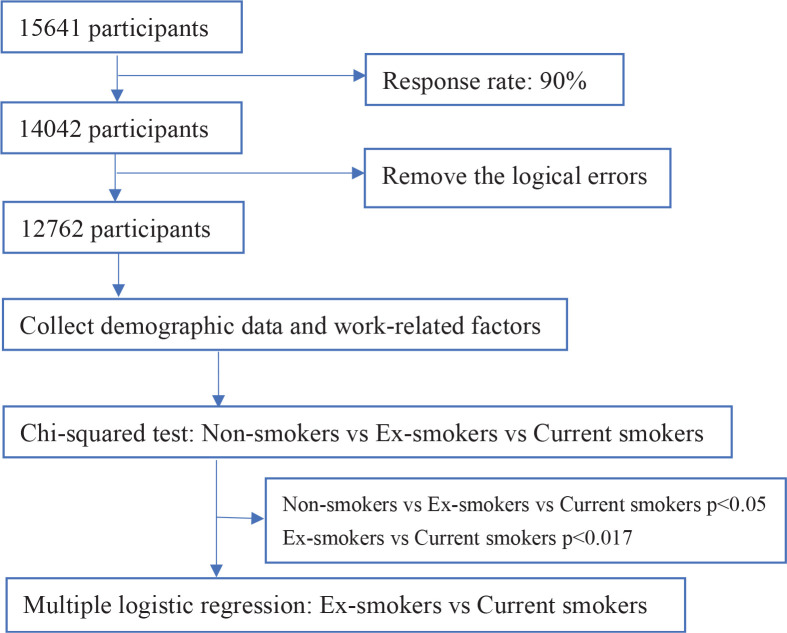
Flow chart of study participant selection and data analysis

This cross-sectional study was part of the National Health Commission-supported China Healthcare Improvement Initiative (CHII), which aimed at improving the health and well-being of medical staff and the quality of care in China’s tertiary hospitals. A total of 41 tertiary psychiatric hospitals in 29 provinces across China participated in the survey from January to March 2021. The target population for the survey comprised psychiatric professionals who were currently employed and willing to participate in the questionnaire survey. Since Gansu Province and the Tibet Autonomous Region did not have provincial-level tertiary psychiatric hospitals, they were excluded from the investigation. CHII intentionally selected tertiary psychiatric hospitals in all provinces of mainland China, as they represent the top-tier hospitals in the nation. The survey was conducted anonymously through WeChat, an online social media application widely used in China. To avoid duplicate registration, only one questionnaire could be submitted for each WeChat account. The questionnaires with logical errors were excluded, including those with a difference of less than 0 years (?) between work experience and night shift experience, more than 1000 beds managed per month, more than 1500 outpatients visit per month, monthly income below 1000 or exceeding 100000 RMB (1000 Chinese Renminbi about US$140), a difference of less than 10 years between age and smoking experience, and daily smoking exceeding 100 cigarettes. After excluding logical errors, 12762 valid responses were included in the statistical analysis (Figure 1).

The study was approved by the Ethics Committee of Chaohu Affiliated Hospital of Anhui Medical University. Informed consent was given by default when the complete questionnaire was submitted.

### Questionnaire design

We used a WeChat-based questionnaire to collect demographic data, including age, sex, education level (associate degree or less, college degree, Master’s degree or higher), marital status (married, single, divorced or widowed), region (Eastern, Central, Western or Northeastern China), and work-related factors including professional title and job satisfaction. Age groups were categorized according to the World Health Organization criteria for designating age groups (20–34, 35–49, ≥50 years). Job satisfaction was measured using the abbreviated form of the Minnesota Satisfaction Questionnaire, with scores ranging from: 1 = ‘very dissatisfied’ to 5 = ‘very satisfied’. A score of ≥80 indicated satisfaction^20^.

Health-related behaviors, such as physical exercise, and alcohol use, were also recorded. ‘Regular exercise’ was defined as engaging in exercising at least 3 times per week, following the recommendations of the National Fitness Guideline. ‘Alcohol use’ was defined as having consumed alcohol at least twice a month in the past year.

The Maslach Burnout Inventory-Human Services Survey (MBI-HSS) was used to measure burnout^21^. It is a 22-item scale that rates burnout in the following three domains: emotional exhaustion (EE), which involves nine items; depersonalization (DP), which consists of five items; and reduced personal accomplishment (PA), which involves eight items. The items were scored on a 7-point scale from 0 to 6, depending on the frequency of symptoms. Participants with high emotional expression (≥27) and/or DP (≥10) scores were defined as suffering from ‘burnout’.

Smoking habits were assessed through a question about current smoking status that had three responses: non-smokers, ex-smokers, and current smokers. ‘Smoking’ was defined as having smoked at least 100 cigarettes, and ‘ex-smoker’ was defined as having ceased smoking for more than three months before the survey^22^. Both current smokers and former smokers were classified as lifetime smokers.

### Statistical analysis

Numerical data were converted into categorical variables and sample distributions were calculated as frequencies and percentages. Chi-squared tests were performed to initially screen for factors associated with smoking cessation, comparing ex-smokers with current ones. A Bonferroni corrected significance level of 0.017 (0.05/3) was applied. Factors from the initial screening were then subjected to adjusted binary logistic regression analysis (Method=Forward: LR) to identify independent factors for smoking cessation. The regression model’s goodness of fit was assessed using the Hosmer and Lemeshow test. For each variable, the beta coefficient, odds ratio, and 95% confidence interval were calculated, along with the corresponding p-value. SPSS 23.0 software was used for all statistical analyses, with a two-tailed p<0.05.

## RESULTS

### Demographic characteristics of participants

A total of 12762 psychiatric professionals, including 3722 psychiatrists (29.2%) and 9040 psychiatric nurses (70.8%), participated in the survey and were included in the statistical analysis. Table 1 shows the characteristics of the psychiatric professionals. Of these psychiatric professionals, 1104 were non-smokers, 1196 were current smokers, and 462 were ex-smokers, for an ex-smoker prevalence of 3.6%. About three-quarters (73.2%) of the total sample were female, more than half (51.2%) were aged <35 years and 11.1% had a Master’s degree or higher. In the whole sample, the rates of burnout, regular exercise, job satisfaction, and alcohol use were 26.8%, 6.5%, 25.7%, and 38.8%, respectively.

**Table 1:** Basic characteristics of psychiatric professionals by smoking status (N=12762)

*Characteristics*	*All (N=12762) n (%)*	*Smoking status*			*χ^2^*	*p_0_*	*p_1_*
		*Non-smokers (N=11104) n (%)*	*Ex-smokers (N=462) n (%)*	*Current smokers (N=1196) n (%)*			
**Profession**					27.592	<0.001	0.268
Psychiatrists	3722 (29.2)	3150 (28.4)	169 (36.6)	403 (33.7)			
Psychiatric nurses	9040 (70.8)	7954 (84.6)	293 (63.4)	793 (66.3)			
**Age** (years)					73.330	<0.001	<0.001
20–34	6539 (51.2)	5763 (51.9)	196 (42.4)	580 (48.5)			
35–49	4768 (37.4)	4166 (37.5)	166 (35.9)	436 (36.5)			
≥50	1455 (11.4)	1175 (10.6)	100 (21.6)	180 (15.1)			
**Sex**					3898.384	<0.001	0.004
Male	3425 (26.8)	1930 (17.4)	397 (85.9)	1098 (91.8)			
Female	9337 (73.2)	9174 (82.6)	65 (14.1)	98 (8.2)			
**Marital status**					10.267	0.036	0.023
Single	2684 (21.0)	2324 (20.9)	87 (18.8)	273 (22.8)			
Married	9863 (74.9)	8341 (75.1)	361 (78.1)	361 (72.0)			
Divorced or widowed	515 (4.0)	4339 (4.0)	14 (3.0)	14 (5.2)			
**Professional title**					60.950	<0.001	0.027
Junior	6480 (50.8)	5551 (50.0)	239 (51.7)	690 (57.7)			
Middle	4424 (34.7)	3979 (35.8)	127 (27.5)	318 (26.6)			
Senior	1858 (14.6)	1574 (14.2)	96 (20.8)	188 (15.7)			
**Education level**					158.817	<0.001	0.002
Associate degree or lower	2771 (21.7)	2229 (20.1)	140 (30.3)	402 (33.6)			
College degree	8580 (67.2)	7576 (68.2)	275 (59.5)	729 (61.0)			
Master’s degree or higher	1411 (11.1)	1299 (11.7)	47 (10.2)	65 (5.4)			
**Region**					93.003	<0.001	0.001
East China	5170 (40.5)	4590 (41.3)	193 (41.8)	387 (32.4)			
Central China	2611 (20.5)	2283 (20.6)	85 (18.4)	243 (20.3)			
West China	3560 (27.9)	3093 (27.9)	124 (26.8)	343 (28.7)			
Northeast China	1421 (11.1)	1138 (10.2)	60 (13.0)	223 (18.6)			
**Regular exercise**					41.432	<0.001	0.033
No	11822 (93.5)	10327 (94.0)	405 (87.7)	1090 (91.1)			
Yes	822 (6.5)	659 (6.0)	57 (12.3)	106 (8.9)			
**Alcohol use**					1110.594	<0.001	<0.001
No	7804 (61.2)	7404 (66.7)	141 (30.5)	259 (21.7)			
Yes	4958 (38.8)	3700 (33.3)	321 (69.5)	937 (78.3)			
**Job satisfaction**					14.055	0.001	0.024
No	9476 (74.3)	8194 (73.8)	340 (73.6)	942 (78.8)			
Yes	3286 (25.7)	2910 (26.2)	122 (26.4)	254 (21.2)			
**Burnout**					72.220	<0.001	0.002
No	9346 (73.2)	8263 (74.4)	329 (71.2)	754 (63.0)			
Yes	3416 (26.8)	2841 (25.6)	133 (28.8)	442 (37.0)			

p_0_: non-smokers vs ex-smokers vs current smokers. p_1_: ex-smokers vs current smokers. East China: Beijing, Tianjin, Hebei, Shanghai, Jiangsu, Zhejiang, Fujian, Shanghai, Guangdong, Hainan. Central China: Shanxi, Anhui, Jiangxi, Henan, Hubei, Hunan. West China: Inner Mongolia, Guangxi, Chongqing, Sichuan, Guiyang, Yunnan, Shanxi, Qinghai, Ningxia, Xinjian. Northeast China: Liaoning, Jilin, Heilongjiang.

### Factors associated with tobacco smoking among psychiatric professionals

We performed a chi-squared test between the three groups of non-smokers, current smokers, and ex-smokers (Table 1). Significant group differences were found in several sociodemographic, health behaviors and occupational characteristics, including professional (χ^2^=27.592, p<0.001), age group (χ^2^=73.330, p<0.001), sex (χ^2^=3898.384, p<0.001), marital status(χ^2^=10.267, p=0.036), professional title (χ^2^=60.950, p<0.001), geographical region (χ^2^=93.003, p<0.001), education level (χ^2^=158.817, p<0.001), regular exercise (χ^2^=41.432, p<0.001), alcohol use (χ^2^=1110.594, p<0.001), job satisfaction (χ^2^=14.055, p=0.001) and burnout (χ^2^=72.220, p<0.001).

### Factors influencing smoking cessation among psychiatric professionals

We then conducted a between-group comparison between the ex-smokers and current smokers at a test level of α=0.017 (Table 1), and found significant differences in age group, sex, education level, drinking, region, and burnout between these two groups. To explore factors related to smoking cessation among psychiatric professionals in psychiatric hospitals, we then conducted a binary logistic regression analysis on the above differential variables. The test for multicollinearity between the independent variables indicated no issues, with the variance inflation factor (VIF) for all variables <10. The model’s goodness of fit was assessed using the Hosmer and Lemeshow test, resulting in a non-significant p=0.389, indicating a high fit degree of the model.

**Figure 2:**
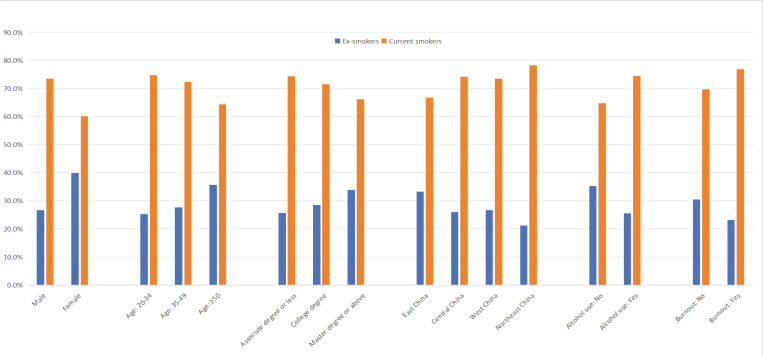
The percentages of ex-smokers and current smokers among lifetime smokers across gender, age group, education level, region, alcohol use, and burnout (N=1658)

Figure 2 shows the percentages of ex-smokers and current smokers among lifetime smokers across sex, age group, education level, region, alcohol use, and burnout.

The logistic regression results (Table 2) revealed the following factors associated with smoking cessation: sex, age group, region, education level, drinking, and burnout. Women had a higher prevalence of ex-smokers than men (AOR=1.89; 95% CI: 1.332–2.666, p<0.001). Individuals in Central China (AOR=0.71; 95% CI: 0.521–0.964, p=0.028) and Northeast China (AOR=0.57; 95% CI: 0.404–0.806, p=0.001) had a lower prevalence of ex-smokers compared to those in East China. Professionals aged ≥50 years had a higher prevalence of ex-smokers than those aged 20–34 years (AOR=1.45; 95% CI: 1.068–1.964, p=0.017). Those holding a Master’s degree or higher had a higher prevalence of ex-smokers than those with an associate degree or lower (AOR=2.17; 95% CI: 1.394–3.366, p=0.001). Individuals who reported drinking had a lower prevalence of ex-smokers than those who did not (AOR=0.65; 95% CI: 0.508–0.837, p=0.001). Notably, compared to current smokers, ex-smokers reported a significantly lower prevalence of burnout (AOR=0.70; 95% CI: 0.552–0.892, p=0.004).

**Table 2:** Multiple logistic regression of factors associating with smoking cessation among psychiatric professionals (N=1658)

*Variables*	*p*	*AOR*	*95 % CI*
**Sex** (Ref. male)	<0.001	1.88	1.332–2.666
**Age** (years) (Ref. 20–34)			
35–49	0.954	0.99	0.772–1.276
≥50	0.017	1.45	1.068–1.964
**Education level** (Ref. associate degree or lower)			
College degree	0.197	1.17	0.920–1.499
Master’s degree or higher	0.001	2.17	1.394–3.366
**Region** (Ref. East China)			
Central China	0.028	0.71	0.521–0.964
West China	0.053	0.76	0.576–1.003
Northeast China	0.001	0.57	0.404–0.806
**Alcohol use** (Ref. No)	0.001	0.65	0.508–0.837
**Burnout** (Ref. No)	0.004	0.70	0.552–0.892

AOR: adjusted odds ratio. Multiple logistic regression adjusted for sex, age group, education level, geographical region, alcohol use, and burnout. Hosmer and Lemeshow test: p=0.389.

## DISCUSSION

This survey marks the first nationwide exploration into factors influencing smoking cessation among psychiatric professionals, addressing an important gap in the existing literature. Drawing from a substantial, nationwide sample comprising psychiatrists and psychiatric nurses, the study found that 9.4% were current smokers, with 3.6% being former smokers who successfully quit. Notably, 462 (27.9%) of 1658 smokers were ex-smokers, which is similar to the previous study^6^. Our study identified several factors associated with smoking cessation in this sample, such as sex, age group, education level, region, drinking, and burnout. Among these factors associated with smoking cessation, region, and burnout are also new findings.

### Gender difference and smoking cessation

A study from Norway found that men were more likely to report having quit smoking to improve their health and physical appearance^23^. However, a study involving population data from the United States, Canada, and the United Kingdom found that the pattern of gender differences in smoking cessation was consistent across countries: women aged <50 years were more likely than men to quit smoking completely, while men were more likely than women to quit at an older age. Across all age groups, there were relatively small differences in smoking cessation between men and women^24^. This suggests a need for prospective randomized studies to understand cultural nuances. Notably, our study focused on Chinese psychiatric professionals and found a two-fold higher prevalence of women ex-smokers compared to men, emphasizing the potential need for gender-tailored interventions.

### Age group and smoking cessation

Consistent with prior research, we found a higher prevalence of ex-smokers among those aged ≥50 years compared to the 20–34 age group^25^. Older smokers are more likely to have smoking-related health problems. They often visit medical institutions for the treatment of diseases, so they are more likely to get smoking cessation advice from medical professionals^25^. The proportion of smokers who quit was low among those aged 35–50 years. This finding suggests health professionals and public health campaigns could emphasize the benefits of quitting by the age of 35 years, to encourage younger smokers to make efforts to quit^26^. A prospective study of one million women in the UK found that those aged <40 years who quit smoking could avoid more than 90% of the excess mortality caused by continued smoking, while those quitting before the age of 30 years could avoid over 97% the excess mortality^11^. Of course, quitting smoking is beneficial for smokers of any age.

### Education level and smoking cessation

An intervention trial in Guangzhou, China, found that the quit rate among smokers was linked to the smokers’ education level^27^. People with college degrees and those with an education level of high school or higher are more likely to quit smoking. In our sample, we found that those with a Master’s degree or higher were more likely to quit smoking than those with a lower level of education. The link between smoking cessation rates and higher education level may be related to awareness of tobacco-related harms, better access to information on tobacco-related treatments, and stronger willingness to quit^28^.

### Alcohol use and smoking cessation

Alcohol and tobacco use are often co-occurring, and alcohol consumption can interfere with successful smoking cessation^15^. Previous studies have shown that the prevalence of smoking is higher among individuals with mild or moderate alcohol use disorder (AUD) than those without AUD, and the severity of AUD is positively correlated with the frequency of smoking^29^. Our study confirmed lower cessation rates among alcohol drinkers than non-drinkers (25.5% vs 35.3%, p<0.001). Moreover, alcohol and tobacco have a synergistic effect on carcinogenicity and other harms^30^; therefore, interventions targeting both tobacco and alcohol use are needed for this population. Quitting smoking can also improve alcohol outcomes in alcohol-dependent individuals, and smoking cessation programs may be beneficial for alcohol treatment programs to support or facilitate abstinence from alcohol^31^.

### Geographical regions and smoking cessation

Regional variations in smoking prevalence have long been recognized in China, including in general populations^32^, and specific groups like psychiatric professionals^6^. The Report on the Current Situation of Smoking in China in 2016, for example, found greater numbers of tobacco smokers in the Central and Western regions and Yunnan Province, followed by the Eastern coastal areas^32^. Similarly, a national survey of psychiatrists found that smoking rates were higher in West and Northeast China than those in East China^6^. Our study adds a new layer to this picture by showing a significant association between smoking cessation and region, with lower rates in Central and Northeastern China compared to Eastern China. This regional disparity can be attributed to several factors. China’s long history of smoking culture, with distinct regional variations like Northeast tobacco and Hunan tobacco, plays a role. Additionally, the central and western regions’ tobacco industries, interwoven with the regional economy, may influence smoking and smoking cessation behavior and local cultures. Finally, the stringency of anti-smoking policies and regulations varies from region to region. The eastern region, being more socioeconomically developed, is known for stricter and better-enforced smoke-free policies, contributing to a decline in smoking prevalence compared to other regions^33^. These insights reveal the significant impact of diverse regional contexts on smoking behaviors and highlight the need for tailored cessation strategies that address the specific challenges of each region.

### Burnout and smoking cessation

Burnout is defined as “a syndrome conceptualized as resulting from chronic workplace stress that has not been successfully managed” according to the 11th revision of the International Classification of Diseases (ICD-11)^34^. Previous studies have found that psychiatric professionals experiencing burnout are more likely to become smokers^6,35^. We found those experiencing burnout had a lower prevalence of ex-smokers. This highlights a complex interplay between stress, coping mechanisms, and smoking cessation. This association may stem from the close connection of burnout to occupational stress and negative emotions^35^, which are known drivers of smoking initiation and major barriers to quitting smoking or risk factors for relapse after quitting^35,36^.

### Strengths and limitations

A few strengths of this study need to be mentioned: 1) national scope: the study covered 41 tertiary psychiatric hospitals across 29 provinces, providing a comprehensive and diverse representation of psychiatric professionals; 2) large sample size: the inclusion of 12762 participants enhances the statistical robustness of the findings; and 3) the inclusion of additional data on regions and burnout alongside demographic information, which enrich the study’s potential applications, contributing valuable insights for designing smoking cessation guidelines, enhancement programs, and policies – a research area that has garnered increased attention in recent years.

This study has some limitations that should be acknowledged. First, as a cross-sectional survey, it is hard to infer any causality from the findings. Second, as with all online surveys, self-selection bias may exist and they may have affected the results. However, the response rate of this study was 90%, which may have minimized the issue. Third, due to the presence of limited covariates, residual confounding emerges, restricting the generalizability of our findings to other countries or medical professionals. Finally, lack of pre-testing of the measurement tools to check for any changes/amendments as well as internal reliability especially if using new or newly combined tools and administering them online. In addition, we did not take into account the impact of awareness regarding the health risks of smoking, nicotine dependence levels, and the smoke-free policy in the workplace on smoking cessation.

## CONCLUSIONS

This study is a preliminary exploration of the potential factors associated with smoking cessation among psychiatrists and psychiatric nurses in China. The findings in this study indicate that gender (males), age (younger age group), education (lower level of education), region (Central and Northeastern regions), alcohol consumption, and burnout are shown to be associated with a lower prevalence of ex-smokers. These results may inform public health policymakers to design and implement effective smoking control and cessation strategies for this population. More longitudinal studies using validated questionnaires or objective indicators of smoking are needed to clarify the relationship between smoking cessation and these factors among Chinese psychiatric professionals.

## Data Availability

Data sharing does not apply to this article, but raw data supporting the conclusions of this study will be provided by the authors without reservation.
